# Exoskeleton robotics: from rigid structures to bio-integrated systems

**DOI:** 10.3389/fnbot.2026.1850367

**Published:** 2026-06-12

**Authors:** Liancheng Zheng, Rizauddin Ramli, Wenfeng Zhang

**Affiliations:** 1 School of Mechanical Engineering, Shandong Huayu University of Technology, Dezhou, China; 2 Department of Mechanical and Manufacturing Engineering, Faculty of Engineering and Built Environment, Universiti Kebangsaan Malaysia, Bangi, Selangor, Malaysia

**Keywords:** bio-integrated, exoskeleton robotics, human-robot interaction, rehabilitation engineering, soft robotics, wearable robotics

## Abstract

Exoskeleton robots have become a representative class of wearable robotic systems for rehabilitation, mobility assistance, occupational support, and human performance augmentation. As the field moves from laboratory prototypes toward clinical, industrial, and daily life deployment, research priorities are shifting from device-centered performance improvement to human-centered integration. This mini review provides a structured and critically oriented synthesis of exoskeleton technologies from four interconnected perspectives: technical architecture, technological paradigm evolution, deployment barriers, and future research directions. To improve transparency and reproducibility, we adopted a narrative review strategy with explicit literature selection criteria. Publications were identified from major scientific databases using combinations of keywords related to exoskeleton robotics, actuation, control, human-robot interaction, soft robotics, neural interfaces, rehabilitation, and wearable assistance. Representative studies were selected according to relevance, technical influence, clinical or engineering significance, and coverage of major technological paradigms. The review first analyzes three core technical dimensions-actuation systems, control strategies, and human-robot interaction which jointly determine the performance, adaptability, and usability of exoskeleton systems. Rather than only summarizing these technologies, we compare their trade-offs in terms of power density, control precision, compliance, energy efficiency, personalization, safety, and deployment readiness. The review then examines the evolution from rigid exoskeletons, which provide high structural support and precise force transmission, to soft exoskeletons, which improve compliance and comfort, and further to bio-integrated systems that combine neural interfaces, functional electrical stimulation, multimodal sensing, and mechanical assistance. Based on this synthesis, we organize the review using a Human–Exoskeleton Integration Maturity Framework spanning mechanical coupling, physical compliance, functional adaptation, and cognitive/bio-integrated coupling. Persistent barriers, including energy supply, personalization, safety assurance, cost, regulatory translation, and ethical governance, are critically discussed. Finally, future directions are outlined, including neural-interface-driven control, multimodal perception, human-in-the-loop optimization, hybrid rigid-soft architectures, and socially responsible design. Overall, this review argues that the next stage of exoskeleton development will depend not merely on stronger actuators or more intelligent algorithms, but on integrated systems that are adaptive, trustworthy, affordable, and seamlessly embedded in human movement and function.

## 1 Introduction

Exoskeleton robots have attracted increasing attention as wearable robotic systems for rehabilitation, physical assistance, and human performance augmentation. With the gradual transition from prototype development to real-world use, the focus of exoskeleton research has expanded from improving power and motion support to enhancing human-centered compatibility. As a result, current studies increasingly emphasize not only mechanical output, but also the integration of actuation, control, and human-robot interaction to achieve safe, adaptive, and effective assistance.

The concept of exoskeletons dates back to the 1890 patent for a “walking assistance device” by Russian engineer Yagn, whose core principle — transferring loads from limbs to the ground — remains foundational to structural design today (Yagn, 1890). However, modern exoskeleton research began in earnest with the U.S. military-led Hardiman project in the 1960s. Though this 680-kg system failed to achieve practicality due to control and energy constraints, it established the technical agenda for the next half-century: power, control, and human-machine coupling.

Functionally, exoskeletons are categorized into three application domains: rehabilitation training (Colombo et al., 2000), performance augmentation (Walsh et al., 2006), and assistive substitution (Esquenazi et al., 2012). Recent rapid adoption of industrial exoskeletons in logistics and material handling signals a maturation of technical readiness. Concurrently, research focus is shifting from “machine-centric” to “human-centric” design -moving from “humans adapting to machines” to “machines understanding humans” (Bosch et al., 2016; Ferris and Sawicki, 2017).

Existing review articles on exoskeleton robotics have provided valuable insights into specific subsystems, including actuation, control, rehabilitation applications, soft exosuits, and neural interfaces. However, many of these reviews remain subsystem-specific and do not fully explain how structural design, actuation choice, control architecture, human–robot interaction, and bio-integration jointly shape exoskeleton evolution and application readiness. Consequently, there remains a need for a cross-domain review that not only summarizes the field, but also interprets exoskeleton development through the lens of design trade-offs and human-centered integration.

Recent review studies have also become increasingly rigorous, particularly in areas such as advanced actuation technologies, artificial muscles, and structured benchmarking of exoskeleton components. These studies have substantially improved subsystem-level understanding and emphasized transparency, reproducibility, and performance-oriented comparison. In contrast, the present mini-review is intended to connect subsystem-level advances to the broader progression of exoskeleton systems from rigid mechanical support to soft, adaptive, and bio-integrated platforms. This cross-domain synthesis defines the main scientific positioning of the present manuscript and distinguishes it from subsystem-specific reviews. In particular, recent reviews have provided more focused analyses of advanced actuation technologies, artificial-muscle-based systems, and structured benchmarking of exoskeleton components. The present review differs from these studies by emphasizing a broader cross-domain synthesis that connects subsystem-level advances to technological paradigm evolution, deployment readiness, and human-centered integration.

**Figure 1:**
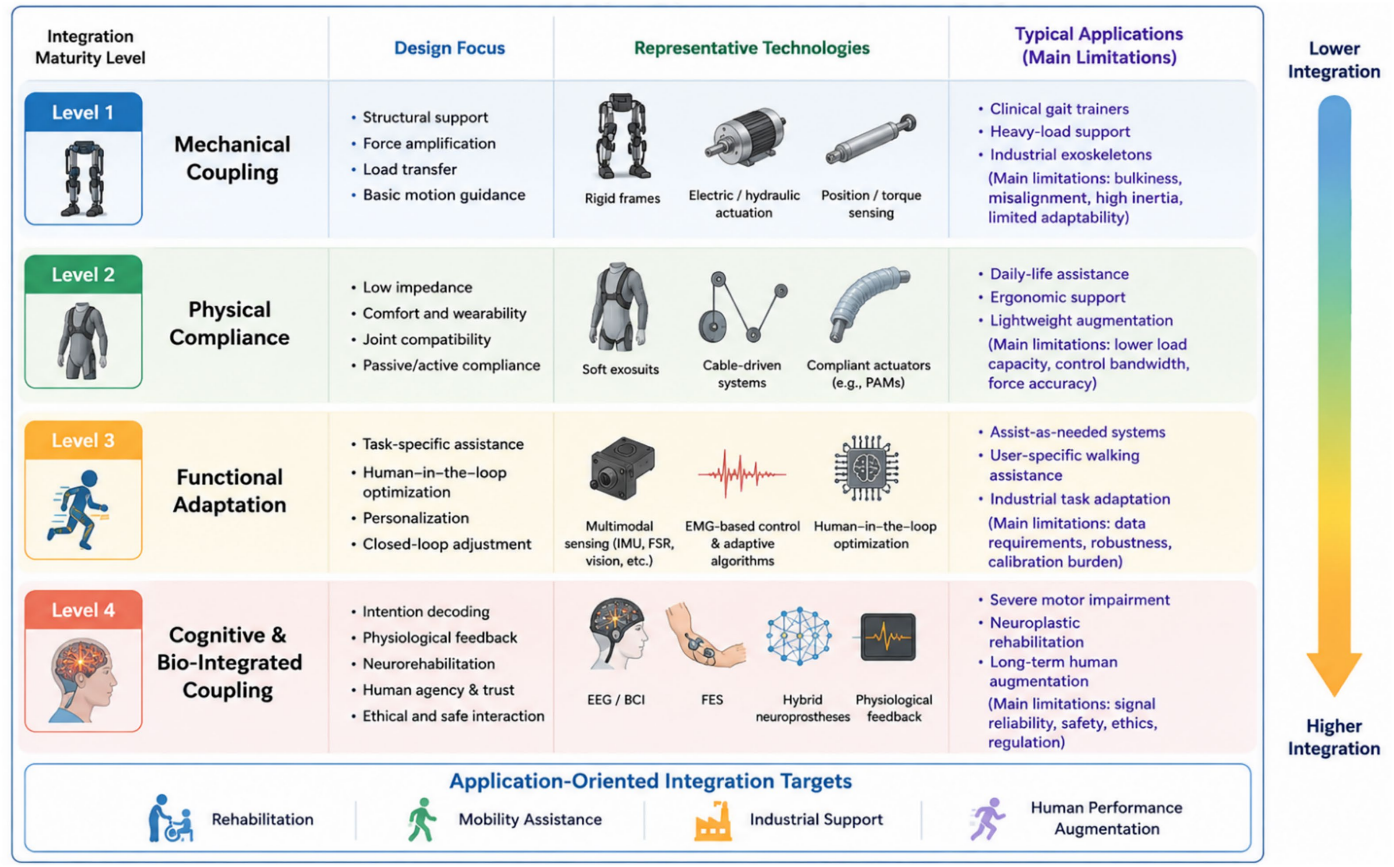
Human–exoskeleton integration maturity framework (HEIMF).

Figure 1 serves as the main analytical map of this review and is used throughout the following sections to relate subsystem choices to different levels of human–exoskeleton integration. To address this gap, the present review provides a structured synthesis of exoskeleton robotics from four perspectives: technical architecture, technological paradigm evolution, deployment barriers, and future research directions. As summarized in Figure 1, the reviewed literature is interpreted using the Human–Exoskeleton Integration Maturity Framework (HEIMF), which spans mechanical coupling, physical compliance, functional adaptation, and cognitive/bio-integrated coupling. The contribution of this review is threefold: first, it compares key technologies using engineering and human-centered evaluation criteria rather than purely descriptive categories; second, it uses the HEIMF to explain why exoskeletons are evolving from rigid to soft and bio-integrated systems; and third, it links subsystem-level design choices to clinical, industrial, and daily-life deployment requirements.

The review was conducted as a structured narrative mini-review rather than a full systematic review or meta-analysis. Representative studies were identified through major scientific databases and were selected according to relevance, technical influence, and coverage of major technological paradigms. A concise description of the literature identification, selection, and synthesis strategy is provided in Section 2.

## 2 Review methodology and analytical framework

This article is designed as a structured narrative mini-review rather than a full systematic review or meta-analysis. Its purpose is to synthesize representative and influential studies that illustrate major technological paradigms, design trade-offs, and future directions in exoskeleton robotics. To improve transparency, the review followed an explicit literature identification, selection, and synthesis strategy. In addition, the reviewed literature was interpreted using a Human-Exoskeleton Integration Maturity Framework (HEIMF), which serves as the main analytical lens of this study.

### 2.1 Literature identification and synthesis strategy

Relevant publications were identified through major scientific databases, including Web of Science, Scopus, IEEE Xplore, PubMed, ScienceDirect, and SpringerLink. Google Scholar was used as a supplementary source to cross check potentially relevant publications and recent review articles. The search included combinations of keywords related to exoskeleton robotics, wearable robots, rehabilitation exoskeletons, soft exosuits, human-robot interaction, exoskeleton actuation, exoskeleton control, EMG control, BCI, neural interfaces, functional electrical stimulation, energy efficiency, safety, and personalization. The primary literature search covered publications from 2016 to 2025, while earlier seminal studies were selectively included when necessary to explain the historical and conceptual development of exoskeleton systems.

Studies were included if they introduced representative exoskeleton systems, provided important technical insights into actuation, control, sensing, human–robot interaction, or bio-integration, reported clinical, biomechanical, ergonomic, or performance outcomes, or addressed major deployment barriers such as safety, energy supply, cost, personalization, and ethics. Studies were excluded if they were unrelated to wearable robotic assistance, lacked sufficient technical or clinical detail, duplicated findings already represented by more authoritative publications, or focused only on non-wearable robotic platforms.

The selected literature was synthesized using a thematic and comparative approach. First, studies were grouped according to three core technical dimensions: actuation systems, control strategies, and human–robot interaction. Second, exoskeleton development was organized into three major technological paradigms: rigid exoskeletons, soft exoskeletons, and bio-integrated exoskeletons. Third, different technologies were compared according to design-relevant criteria, including force transmission, power density, compliance, control precision, energy efficiency, personalization, safety, cost, and technology readiness. Because exoskeleton studies differ substantially in target populations, task conditions, device configurations, and evaluation protocols, direct quantitative comparison is often difficult. Therefore, this review adopts a quantitatively informed comparative approach, in which representative indicators such as device mass, power requirements, metabolic cost reduction, control latency, assistance torque, and system cost are discussed where available, while acknowledging heterogeneity across studies.

### 2.2 Human–exoskeleton integration maturity framework (HEIMF)

To strengthen the analytical perspective of this review, the selected literature was interpreted using a Human-Exoskeleton Integration Maturity Framework (HEIMF). This framework conceptualizes exoskeleton development in terms of progressively deeper forms of human–machine integration and helps explain why exoskeletons are evolving from rigid mechanical systems toward soft, adaptive, and bio-integrated platforms.

At level 1: Mechanical Coupling, the exoskeleton primarily functions as an external mechanical support or force amplification device. The main design priorities are structural strength, torque generation, and load transfer. Representative systems include rigid clinical gait trainers and early load-carrying exoskeletons. Their main advantages are high force transmission and repeatable trajectory control, whereas their main limitations include kinematic mismatch, high inertia, and limited comfort.

At level 2: Physical Compliance, the design emphasis shifts toward mechanical compatibility with the human body. Soft exosuits, textile-based structures, cable-driven systems, and compliant actuators are representative technologies at this level. The main objective is to reduce motion interference, improve comfort, and lower mechanical impedance. However, this increased compliance often comes at the cost of reduced force precision and load-bearing capability.

At level 3: Functional Adaptation, the exoskeleton begins to adjust assistance according to user-specific movement patterns, fatigue states, and task requirements. Human-in-the-loop optimization, learning-based control, adaptive impedance control, and multimodal sensing become increasingly important. At this level, the device no longer provides fixed assistance, but dynamically adapts its behavior based on user response and task context.

At level 4: Cognitive and Bio-Integrated Coupling, the exoskeleton moves beyond mechanical assistance toward intention-aware and physiologically informed interaction. User intention and physiological states may be decoded through EMG, EEG, peripheral nerve signals, brain–computer interfaces, or other biosignals. In bio-integrated systems, exoskeleton assistance may also be combined with functional electrical stimulation, neurofeedback, and closed-loop rehabilitation strategies. The design goal is not only to assist motion, but also to participate in motor control, functional restoration, and neuroplastic rehabilitation.

The HEIMF provides a useful interpretive lens for understanding the transition from rigid to soft and bio-integrated exoskeletons. These four levels should not be interpreted as strictly linear stages, but as analytically distinct dimensions that may coexist within hybrid systems. In practice, future exoskeletons are likely to combine structural support, compliance, adaptive control, and neural integration according to application-specific requirements in rehabilitation, assistance, industrial support, and human augmentation.

## 3 Three dimensions of technical architecture

The three technical dimensions discussed below are compared using recurring design criteria, including power density, control precision, compliance, energy efficiency, control bandwidth, safety, adaptability to user variability, and deployment readiness.

### 3.1 Actuation systems: trade-offs between energy, power, and compliance

The actuation system is the “muscle” of an exoskeleton, directly determining its force-to-weight ratio, response speed, and safety. Current exoskeleton actuation technologies include conventional rigid actuators, compliance-oriented actuators, and emerging soft or bio-inspired actuation concepts, although these categories increasingly overlap in hybrid systems. Electric motor actuation is the most widely adopted technology, valued for high control precision, fast response, and technical maturity (Walsh et al., 2006; Esquenazi et al., 2012). However, it faces an inherent force-to-weight trade-off: high-torque output requires large, heavy motors, creating a vicious cycle of “heavier systems needing more torque, and more torque requiring heavier motors.”

Hydraulic and pneumatic actuation: Hydraulic systems offer superior power density, making them ideal for heavy-load scenarios (Herr, 2009). Yet low energy efficiency, leakage risks, and maintenance complexity limit civilian applications. Pneumatic systems, with inherent compliance, are promising for rehabilitation but suffer from poor controllability and load-bearing capacity.

Flexible and soft actuation: Emerging technologies are reshaping exoskeleton design. Harvard’s soft exoskeleton suit uses cable-driven textile structures with offboard actuation to reduce wearer burden. While these solutions excel in human-machine compatibility, they require breakthroughs in output power, control accuracy, and fatigue life (Tondu and Lopez, 2000). Notably, the three approaches are not mutually exclusive. Hybrid actuation (Pratt and Krupp, 2004) is increasingly adopted to balance performance and safety. Another important distinction concerns on-board versus off-board actuation: on-board systems improve portability but increase carried mass and energy demand, whereas off-board actuation reduces wearer burden at the expense of autonomy and ecological validity. Emerging actuation concepts such as electroactive polymers, dielectric elastomers, and shape memory alloys are attracting attention because of their biomimetic potential and high compliance. However, their current limitations in controllable output, efficiency, durability, and integration readiness still restrict widespread use in wearable exoskeletons.

Design implication: future exoskeletons are likely to adopt hybrid actuation architectures that combine rigid power transmission with compliant interfaces. Such systems can balance precision, comfort, and safety.

### 3.2 Control strategies: from model-driven to human-in-the-loop

The core challenge of control systems is aligning the robot’s motion intent with the user’s autonomous movement a task far more complex than in industrial robotics, where humans are external disturbances rather than dynamic system components.

Model-based control was the dominant paradigm in early systems, relying on human biomechanical and exoskeleton dynamic models to achieve trajectory tracking (Colombo et al., 2000). However, model-based methods are sensitive to parameter variations and modeling errors, which limits their robustness under inter-user variability, fatigue, and changing task conditions, even though their computational structure is usually more interpretable and easier to validate in real time.

Learning-based control: Reinforcement learning enables exoskeletons to adaptively optimize assistance strategies through human-robot interaction. Studies show that learning-based controllers can adapt to individual gait characteristics and improve personalization, but their practical deployment still faces challenges in data efficiency, generalization across users and tasks, safe exploration, and real-time reliability (Zhang et al., 2024).

Biological signal-driven control represents a fully “human-in-the-loop” paradigm that decodes user intent in real time via electromyography (EMG), electroencephalography (EEG) (Farina and Negro, 2015), or mechanical signals. EMG control is the most mature path, though electrode displacement, muscle fatigue, and signal crosstalk persist. Brain computer interfaces (BCIs), while conceptually transformative, are limited by low signal-to-noise ratio, long training time, latency, and insufficient robustness for routine real-time deployment (Millán et al., 2010).

A key trend is the evolution of control architectures from hierarchical “perception-decision-execution” to distributed systems. “Tactile dialogue” implicit coordination between humans and machines via force and motion feedback has emerged as a core topic for next-generation control (Pratt and Pratt, 1995).

Therefore, the transition from model-based to learning-based or physiological control should not be interpreted as a simple replacement trend, but as a shift toward hybrid and shared-control architectures that balance interpretability, adaptability, and safety. Design implication: future control strategies should combine biomechanical modeling, sensor fusion, learning-based adaptation, and physiological intention decoding. Robust shared control will be more practical than fully autonomous control, especially in safety-critical rehabilitation and daily-life environments.

### 3.3 Human-robot interaction: from physical interfaces to cognitive coupling

Human-robot interaction (HRI) encompasses two layers: physical and cognitive interaction. Physical interaction concerns not only force transmission and motion compatibility, but also interface mechanics such as pressure distribution, shear loading, attachment stiffness, and misalignment compensation, all of which directly affect comfort, safety, and long-term usability. The kinematic complexity of human joints makes perfect fitting by rigid exoskeletons difficult (Winter, 2009; Zelik and Ferris, 2010). Soft exoskeletons mitigate this via flexible structures but sacrifice force transmission efficiency (Asbeck et al., 2013).

Cognitive interaction centers on how exoskeletons understand and respond to user goals and states. Traditional systems use a “master–slave” model (Zhang et al., 2024), while advanced systems pursue “collaborative” models (Farina and Negro, 2015). This requires multi-modal perception and sensor-fusion architectures in which kinematic data reflects what is being done, physiological signals indicate what is intended, and contextual information helps infer what assistance should be delivered (Kulić and Croft, 2007). Feedback mechanisms, including haptic, visual, and proprioceptive cues, also play an important role in transparency, trust, and user adaptation, but remain insufficiently standardized in current systems.

Human-robot trust is an understudied critical variable: over-assistance causes muscle atrophy and reduced attention, while under-assistance increases metabolic burden and fall risk (Ferris and Sawicki, 2017). Balancing “assistance” and “empowerment” is both a technical and philosophical challenge (Sheridan, 2016). Design implication: human-robot interaction should be evaluated not only by mechanical performance, but also by comfort, trust, cognitive load, user acceptance, and long-term adherence.

## 4 Three paradigm shifts in technological evolution

The evolution from rigid to soft and, more recently, to bio-integrated exoskeletons should be understood not merely as a chronological progression, but as the result of interacting technological, biomechanical, and application-driven pressures. Rigid systems emerged from the need for force amplification and repeatable mechanical support; soft systems developed in response to the limitations of rigid-body alignment, comfort, and wearability; and bio-integrated systems represent a further shift toward intention-aware assistance and neurophysiological coupling. Consequently, no paradigm is universally superior. The preferred architecture depends on application priorities, including load support, trajectory accuracy, comfort, autonomy, personalization, and rehabilitation goals. The following subsections therefore interpret each paradigm not only by representative devices, but also by the technological, biomechanical, and application-driven conditions under which it becomes advantageous or limiting.

### 4.1 First generation: rigid exoskeletons — the mechanical logic of force amplification

Rigid exoskeletons, built on metal or carbon fiber frames with rotational joints, follow the industrial logic of “machines enhancing humans.” They offer structural stability, high force transmission efficiency, and mature technology. Kawamoto and Sankai (2005) proposed a HAL-3 power-assist method based on EMG feedback, validating the real time performance and effectiveness of EMG signals in controlling exoskeletons. Sankai (2010) introduced the ‘cyborg’ design concept of HAL (Hybrid Assistive Limb), emphasizing its force amplification function through human-machine cooperation using bioelectrical signals. Walsh et al. (2006) developed the autonomous, cable-free full-body exoskeleton BLEEX, demonstrating its stability and force transmission efficiency in enhancing human load-carrying capacity. Esquenazi et al. (2012) clinical studies indicate that the ReWalk exoskeleton can assist patients with complete thoracic spinal cord injury in regaining standing and walking abilities, validating its technological maturity.

However, the major limitation of rigid exoskeletons lies in the mismatch between simplified mechanical joints and the complex kinematics of the human body. Even modest alignment errors may increase interface pressure, alter joint loading, reduce comfort, and compromise long-term usability, especially outside tightly controlled rehabilitation settings. Winter (2009) systematically analyzed the biomechanical characteristics of human movement, pointing out that the nonlinearity and high degrees of freedom of human joint motion are difficult to fully simulate using rigid hinge models. Ferris and Sawicki (2017) reviewed the development of exoskeleton technology, noting that long-term use of rigid exoskeletons may lead to gait abnormalities and increased energy consumption. This creates a recurring trade-off between structural rigidity, which improves load support and repeatable guidance, and biomechanical adaptability, which is necessary for natural movement and sustained wear. Zhang by optimizing exoskeleton assistance strategies, found that joint load is closely related to gait abnormalities, and personalized control is needed to mitigate negative effects (Zhang et al., 2017). Additionally, end-limb mass concentration increases movement energy consumption — some studies report higher metabolic cost when wearing rigid exoskeletons. Herr (2009) systematically summarized the classification and design challenges of exoskeletons and orthoses, pointing out the fundamental limitations of rigid structures in adaptability, comfort, and human-machine interaction. Walsh (2018) advocated a “human-centered” exoskeleton control paradigm, emphasizing that future developments need to achieve human-machine collaboration through real-time sensing and adaptive algorithms.

The critical lesson from rigid exoskeletons is that mechanical strength alone does not guarantee functional benefit; support is valuable only when it does not excessively increase metabolic burden, discomfort, or movement constraint. A device can generate high torque but still fail to improve user performance if it increases energy expenditure, reduces comfort, or disrupts natural motor control.

### 4.2 Second generation: soft exoskeletons a flexible human machine compromise

Soft exoskeletons, based on textiles, cables, and flexible actuators, are guided by a core insight: adapt to the human body’s complexity rather than fight it (Collins et al., 2015). Harvard’s soft exoskeleton, a milestone in this field, uses pneumatic artificial muscles and fabric straps, weighing approximately one-third to one-seventh of rigid systems (Asbeck et al., 2013; Walsh et al., 2014). De Rossi and Carpi (2017) proposed that flexible wearable robots achieve natural interaction with the human body through soft materials and biomimetic design, suitable for personal assistance and rehabilitation. Polygerinos et al. (2017) reviewed fluid-driven flexible robot technologies, covering manufacturing, sensing, control, and their potential applications in human-robot interaction. Advantages include low mechanical impedance (no restriction on natural movement), high wear comfort, and lower manufacturing costs. However, compliance comes at the cost of reduced force precision and load capacity, limiting applications to gait assistance and light upper limb tasks. Awad et al. (2020) systematically reviewed the progress of soft exoskeletons in gait assistance, emphasizing their low mechanical impedance and wearing comfort, but noting limitations in force output accuracy. Mazzolai et al. (2012) explored plant- and animal-inspired soft robotics technologies, highlighting their low cost and biocompatibility advantages, suitable for medical and daily assistance. Kang and Jung (2019) reviewed the application of flexible exoskeletons in rehabilitation, pointing out insufficient force output accuracy, which limits their applicability in high-load tasks. Awad et al. (2017a,b) analyzed the limitations of soft exoskeletons, emphasizing that they are more suitable for lightweight assistance tasks (such as gait optimization) rather than high-precision rehabilitation training.

A key question is not whether softness is inherently superior, but under which operating conditions it provides a more suitable compromise than rigid architectures. Comparative studies show rigid structures remain irreplaceable for rehabilitation requiring precise trajectory guidance, while soft solutions dominate daily life assistance. By contrast, in rehabilitation contexts that require repeatable guidance, precise joint-level assistance, or higher structural authority, soft systems may be suboptimal because their compliance can reduce force transmission accuracy and controllable output. Hesse et al. (2006) summarized 10 years of experience from the Berlin team, demonstrating that rigid exoskeletons can provide precise trajectory guidance in stroke rehabilitation, but lack flexibility. Zhang et al. (2017) verified the high-precision control advantages of rigid exoskeletons in gait assistance through human-machine collaborative optimization algorithms, though potentially increasing metabolic cost. Panizzolo et al. (2016) proposed bio-inspired multi-joint soft exoskeletons, significantly reducing the energy consumption of load-bearing walking, highlighting their potential for daily assistance. An emerging hybrid paradigm combines rigid skeletons (for structural support) and soft interfaces (for human-machine compatibility). Kim and Tadesse (2020) explored innovative applications of soft robotics in human-robot interaction, proposing a rigid-flexible integrated design that accommodates both structural support and human body adaptability. Lee et al. (2021) proposed a hybrid exoskeleton framework that enhances movement precision and wearing comfort through the synergy of a rigid skeleton and flexible interfaces. Asbeck et al. (2015) developed a soft exoskeleton for running assistance, balancing support and natural motion compatibility through lightweight design. Walsh (2018) advocated a “human-centered” exoskeleton control paradigm, emphasizing the importance of real-time sensing and adaptive algorithms. Polygerinos designed soft robotic gloves for hand rehabilitation, validating the effectiveness and challenges of flexible actuation in task-specific training (Polygerinos et al., 2015).

A key insight is that rigid and soft exoskeletons should not be interpreted as competing categories in a simple superiority hierarchy. Rather, they occupy different regions of the design space: rigid systems are stronger in alignment and guidance, whereas soft systems are stronger in comfort and unobtrusiveness. Hybrid rigid soft exoskeletons are therefore increasingly attractive because they combine structural support with compliant human interfaces.

### 4.3 Third generation: bio-integrated exoskeletons an integrated approach beyond mechanics

Bio-integrated exoskeletons represent a radical vision: instead of “replacing” or “assisting” human functions, they reconstruct motor control circuits via neural interfaces and FES. The core innovation is integrating exoskeletons, neural interfaces, and FES into a unified system. Herr and Smith (2021) proposed the Hybrid Neural Prosthesis (HNP) framework, achieving synchronized control by integrating neural interfaces with exoskeletons, advancing neuro-machine collaborative rehabilitation technologies. Farina systematically analyzed the decoding methods of surface electromyography (sEMG) signals in upper limb prosthesis control, exploring the challenges of signal extraction and emerging solutions (Farina et al., 2014).

In the hybrid neuroprosthetic (HNP) paradigm, implanted or surface electrodes are used to acquire physiological or neural signals that are subsequently decoded to coordinate exoskeleton assistance and, in some cases, functional electrical stimulation. The practical value of this paradigm depends on signal quality, decoding accuracy, latency, and robustness under real-world conditions. Paralyzed users not only regain walking ability but also experience neural plasticity improvements — long-term use has been shown to promote partial neural function recovery. Courtine et al. (2023) reviewed the clinical progress of brain-spinal cord interfaces in the walking recovery of paralyzed patients, emphasizing the potential of long-term use to promote neural function reconstruction. Herr (2024) demonstrated that bio-integrated exoskeletons synergize with neural interfaces and functional electrical stimulation (FES) to significantly improve neuroplasticity and motor function in paralyzed patients. Although this direction is promising, its current limitations include signal instability, calibration burden, limited robustness across sessions, and the difficulty of translating laboratory-level decoding performance into clinically reliable long-term assistance.

Challenges include long-term neural interface stability, biocompatibility, and ethical scrutiny. Nevertheless, this direction signals a fundamental trend: exoskeletons are evolving from “wearable machines” to “extensions of the nervous system.” Lacour et al. (2010) explored the material and technical bottlenecks of flexible implantable neural prostheses, focusing on addressing issues of long-term stability and biocompatibility. Chen analyzed the limitations of wearable electronic devices in neural signal acquisition and proposed innovative design directions to improve the signal quality of surface electrodes (Chen et al., 2022). Warwick and Shah (2018) discussed ethical controversies arising from implantable neural devices, including privacy, autonomy, and risks of technological misuse. Micera and Navarro (2020) systematically summarizes the progress of bidirectional neural interfaces in prosthetic control and sensory feedback, pointing out the technical and regulatory obstacles that need to be overcome for clinical translation.

The critical insight is that bio-integration changes the role of exoskeletons from wearable mechanical aids toward systems that may participate in neuromuscular control; precisely for this reason, their development must be evaluated with both engineering rigor and clinical realism. They are no longer simply wearable machines but may become extensions of the human neuromuscular system. This transition requires not only engineering innovation but also clinical validation, regulatory governance, and ethical design.

Tables 1, 2 provide a design-oriented synthesis of exoskeleton paradigms and actuation families. Rather than serving as exact benchmark databases, they summarize representative trade-offs, application contexts, and literature-supported trends across heterogeneous studies (see Tables 3, 4).

**Table 1:** Comparative characteristics of three exoskeleton paradigms.

Paradigm	Main design objective	Key advantage	Main limitation	Preferable application context	Representative evidence	Key references
Rigid exoskeletons	Structural support and repeatable force transmission	High load-bearing capacity and precise trajectory guidance	Kinematic mismatch, higher distal mass, reduced comfort	Clinical gait training, heavy-load support, tasks requiring structural authority	Clinical studies on ReWalk and Lokomat-type systems support their value in structured rehabilitation and mobility restoration	Herr, (2009); Esquenazi et al. (2012)
Soft exoskeletons	Compliance, low impedance, and wearable comfort	Better human compatibility, lower mass, improved mobility assistance	Lower force precision and limited structural support	Daily assistance, occupational support, metabolic optimization during walking	Studies have reported measurable reductions in walking metabolic cost under assistance conditions	Walsh et al. (2014); Awad et al. (2020)
Bio-integrated exoskeletons	Intention-aware assistance and neurophysiological coupling	Potential for neurorehabilitation and closer coupling to user intention	Signal instability, calibration burden, long-term translational and ethical challenges	Severe motor impairment, neurorehabilitation, hybrid neural-assistive systems	Brain–spine and hybrid neuroprosthetic studies suggest translational potential beyond purely mechanical assistance	Herr and Smith, (2021); Courtine et al. (2023)

**Table 2:** Design-oriented comparison of representative exoskeleton actuation families.

Actuation family	Typical characteristics	Main limitation	Suitable applications	Design implication	Key references
Electric motor	High control precision, mature integration, compact control architecture	Torque–weight trade-off, transmission friction, battery demand	Rehabilitation, assistive walking, industrial support	Widely used in portable rigid exoskeletons, but increasing output often raises carried mass	Bettella et al. (2025);
Hydraulic	High force and power density	Leakage risk, noise, maintenance complexity, lower portability	Heavy-load and military-oriented systems	Most suitable when force demand dominates over portability and wear comfort	Tiboni et al. (2022); Garofalo et al. (2024)
Pneumatic	High compliance and safer interaction	External air supply, nonlinear control, limited portability	Rehabilitation and soft assistance	Attractive for compliant interaction, but less favorable for untethered daily use	Garofalo et al. (2024); Soni et al. (2025)
Cable-driven/off-board actuation	Low distal mass, remote power transmission	Routing losses, friction, reduced autonomy if off-board	Soft exosuits, experimental gait assistance	Particularly attractive in soft systems, but performance depends strongly on transmission efficiency and interface stiffness	Asbeck et al. (2013); Walsh et al. (2014)
Series elastic actuator	Improved force controllability and impact tolerance	Lower position accuracy, added mechanical complexity	Lower-limb exoskeletons requiring interaction safety	Useful when force control and compliance are prioritized over strict tracking precision	Bettella et al. (2025); Sarkisian et al. (2023)
Soft/bio-inspired actuators	High compliance and body conformity	Lower controllable output, fatigue, limited maturity	Emerging wearable assistance concepts	Promising for future soft exoskeletons, but not yet as mature as motor-driven solutions	Garofalo et al. (2024)

The descriptors reported in Tables 1, 2 are synthesized from representative studies and are intended as design-oriented comparative summaries rather than exact universal benchmarks, because exoskeleton studies differ substantially in task conditions, target populations, device architectures, and evaluation protocols.

**Table 3:** Comparison of Control Strategies.

Control strategy	Core principle	Advantage	Limitation	Suitable scenario
Model-based control	Biomechanical or dynamic modeling	Stable, interpretable, precise	Poor adaptability to users and tasks	Clinical trajectory training
Impedance/admittance control	Regulates interaction force-motion relationship	Safer physical interaction	Parameter tuning is difficult	Rehabilitation and assistive walking
EMG-based control	Decodes muscle activation	Early intent detection	Fatigue, crosstalk, electrode shift	Voluntary assistance
EEG/BCI control	Decodes cortical intention	Potential for severe paralysis	Low SNR, long training, latency	Neurorehabilitation
Learning-based control	Optimizes assistance from data	Personalized adaptation	Data/training burden	Adaptive assistance
Human-in-the-loop optimization	Uses user response as optimization objective	Can reduce metabolic cost	Requires repeated measurement	Walking/running assistance

**Table 4:** Evaluation criteria for exoskeleton design.

Criterion	Engineering meaning	Human-centered meaning	Example metric
Force/torque output	Ability to provide mechanical assistance	Functional support	Peak torque, continuous torque
Mass and inertia	Device weight and distribution	Comfort and metabolic cost	Total mass, distal mass
Energy efficiency	Battery and actuator performance	Runtime and independence	Operating time, power consumption
Control latency	Delay between intent and assistance	Safety and naturalness	Response time
Compliance	Mechanical adaptability	Comfort and safety	Interface pressure, impedance
Personalization	Adaptation to individual differences	Usability and effectiveness	Calibration time, user-specific gain
Safety	Risk mitigation	Injury prevention	Fall prevention, emergency stop
Cost	Manufacturing and maintenance	Accessibility	Unit price, maintenance cost
Trust and acceptance	Predictability and transparency	Long-term adherence	User satisfaction, perceived safety

## 5 Challenges and future directions

### 5.1 Current challenges in real-world deployment

Energy and endurance remain a core deployment bottleneck because autonomy, actuator output, and carried mass are tightly coupled. In practice, increasing battery capacity improves runtime but also raises system weight and distal inertia, whereas lightweight power supplies often fail to support sustained all-day operation. Rahman and O'Dell (2019) analyzed the trade-off between the weight and endurance of exoskeleton batteries and proposed a hybrid energy solution (such as energy recovery and supercapacitors) to improve energy efficiency. Hartley and Rouse (2021) reviewed the potential and limitations of braking energy recovery technologies in exoskeletons, emphasizing the need to optimize energy conversion efficiency to extend endurance.

#### 5.1.1 Personalized adaptation

Individual differences in movement patterns, body size, and neural control make universal strategies inadequate. Transfer learning may enable rapid adaptation to new users with minimal samples, reducing personalized training costs. Zhang et al. (2017) proposed a “human-machine closed-loop” control based on real-time optimization algorithms, achieving dynamic adaptation of the exoskeleton to individual gait and enhancing the assistive effect. Ren and Park (2022) validated the effectiveness of transfer learning in reducing the need for personalized training data, promoting the feasibility of rapid exoskeleton adaptation to new users.

Safety is critical in rehabilitation — fall risks, abnormal force output, and control delays can cause secondary injuries. Redundant sensors, multi-modal monitoring, and emergency braking are standard but increase system complexity. Awad et al. (2017a,b) designed safety mechanisms for the eLEGS exoskeleton, reducing the risk of falls and abnormal force output during rehabilitation through redundant sensors and dynamic control strategies. Chen et al. (2016) proposed multimodal control strategies (such as multi-sensor fusion and emergency braking) to enhance the safety of exoskeletons in complex environments. In addition to hardware faults, safety must also be considered from the perspective of control instability, delayed intent recognition, inappropriate torque delivery, and human injury risk during unexpected interaction events.

#### 5.1.2 Cost and accessibility

Current rehabilitation exoskeletons typically cost between $50,000 and $200,000, limiting access. Low-cost composites and 3D printing may reduce costs, but quality control remains a challenge. Ghanbari and Winkler (2020) quantified the cost of rehabilitation exoskeletons (50,000–200,000), analyzing how insufficient insurance coverage and the need for policy support restrict their widespread adoption. Pons (2021) explored the potential of 3D printing and low-cost composite materials in reducing the manufacturing costs of exoskeletons, while also highlighting challenges in quality control and standardization.

As exoskeletons become more intelligent and bio-integrated, ethical challenges become more important. Neural and physiological data require strong privacy protection. Users must retain agency and control over assistive decisions. Human augmentation applications may raise concerns about fairness, workplace pressure, and unequal access. Regulatory frameworks must address not only mechanical safety but also software reliability, data governance, and long-term accountability.

Taken together, these barriers are tightly coupled: improving autonomy may increase system mass, enhancing personalization may increase calibration burden, and strengthening safety often requires additional sensing and control complexity. Therefore, future progress depends less on solving a single bottleneck than on balancing these constraints at the system level.

### 5.2 Emerging directions for next generation exoskeletons

Future exoskeletons are likely to evolve along several converging directions that directly address the deployment barriers discussed above. A first priority is the development of hybrid rigid soft architectures that combine the structural support and alignment capability of rigid components with the comfort and compliance of soft interfaces. Such configurations may reduce kinematic mismatch while preserving sufficient mechanical authority, and modular designs may further allow clinicians or users to adjust assistance levels according to task-specific requirements.

A second major direction is the integration of multimodal sensing and more robust sensor-fusion frameworks. Next generation systems are expected to combine kinematic, kinetic, EMG, EEG, pressure, inertial, visual, and environmental information in order to improve intention recognition, motion adaptation, and safety monitoring. However, this trend also introduces important methodological challenges, including missing data, sensor drift, noise, inter user variability, and the need for interpretable fusion models in clinical settings. Closely related to this trend is the increasing role of human-in-the-loop and personalized optimization. Rather than optimizing assistance using a single objective, future systems are likely to adopt multi-objective strategies that jointly consider metabolic cost, comfort, stability, muscle activation, fatigue, and user preference, thereby enabling more balanced and individualized assistance.

A third key direction is the advancement of neural interface-driven assistance. Non-invasive EMG- and EEG-based approaches remain attractive because of their relative deployability, while implanted interfaces may offer higher signal quality in selected applications. In either case, the most promising path is likely to be the integration of neural decoding with mechanical sensing, adaptive control, and, where appropriate, functional electrical stimulation, so as to improve robustness and functional relevance. At the same time, future progress will also depend on socially responsible and inclusive design. Exoskeletons should be developed not only for technical performance in laboratory environments, but also for accessibility, affordability, usability, privacy protection, and long-term benefit in real-world contexts. This is particularly important for diverse user groups, including older adults, individuals with neurological disorders, workers, and users in resource-constrained settings. Overall, the most impactful future advances will be those that improve both technical capability and practical adoptability.

## 6 Conclusion

Exoskeleton robotics has evolved from rigid force-amplifying machines to soft compliant devices and, more recently, toward bio-integrated systems that combine mechanical assistance with neural and physiological interfaces. This evolution reflects a fundamental shift in design philosophy: from maximizing isolated hardware performance to optimizing human-machine integration across mechanical, functional, and cognitive levels.

Rigid exoskeletons remain valuable for high-load support and precise rehabilitation guidance, but they are limited by weight, kinematic mismatch, and comfort issues. Soft exoskeletons improve compliance and wearability, but often sacrifice force precision and load-bearing capability. Bio-integrated systems offer the possibility of intention-aware assistance and neuroplastic rehabilitation, but they face significant challenges in signal stability, safety, ethics, and clinical translation.

This review organized the analysis using the Human-Exoskeleton Integration Maturity Framework, which helps explain the progression from mechanical coupling to physical compliance, functional adaptation, and cognitive/bio-integrated coupling. Large-scale adoption will require progress in energy supply, personalization, safety validation, cost reduction, regulatory approval, and ethical governance. Ultimately, the future of exoskeleton robotics lies not in making machines simply stronger or smarter, but in making assistance more adaptive, trustworthy, affordable, and seamlessly embedded in human movement and daily life.
